# Supplementary material for: Barriers and facilitators to social inclusion among people with severe mental illness: A qualitative study
Source: PLOS Ment Health. 2025 Apr 24;2(4):e0000137. doi: 10.1371/journal.pmen.0000137 (PMC12798190; doi:10.1371/journal.pmen.0000137)
Supplement: S1 File — (DOCX) [file pmen.0000137.s001.docx]

**S1 File: Interview topic guides.**

**Topic guide for service user in-depth interview**

**(v.1. 17.09.15)**

[Off- tape ice breaker: remind participant about nature of the in-depth interview - ‘I’m going to ask you a bit about yourself, similar to the questionnaire…and how you think things can be improved…(and practical matters re time - about half hour, tape-recorder, etc), explain you may jot down 2/3 points during interview)

*Hint - use open questions eg ‘what might stop you getting on a course/job etc like that?, if an experience is stated, follow with ‘what do you mean by that, request more detail, can you give an example? etc*

**Start of taped interview**

**General**

**I know I asked you before in our earlier interview, but for the purposes of this recording, could you remind me what your mental health problem is?**

**What is your living situation? Are you working? Do you have children? If not living with children, why?** Note responses to discuss later eg housing, partner, children, work, courses, and friends

*Hint - can signpost - ‘that sounds important - we can come back to that’*

**How did you find the first interview?** Did any of the questions asked provoke any thought? Have they had any thoughts about social inclusion since? Or any topics that came up?

**Explain project and what we understand social inclusion to be**

Leisure activities (hobbies; sports etc), social relationships, access to education/employment, transport, access to health services, housing, etc

**Do you or have you felt excluded in any of these areas? What would need to change for you to feel more socially included? Have you tried to be involved and been stopped from doing so?**

**If yes, in which areas? How important is it to you to feel included in <topic mentioned>**

**If no, in which areas? Do you feel this affects you in some way? How?**

Open discussion of areas in which individual feels included/excluded and the way they feel about it; what stops them if excluded, eg lack of confidence, ability, and mental health revealed etc. If parent, would they like more contact? What prevents them? What do they think reasons are for exclusion in areas mentioned?

**Current support**

**Have you received any support in** **< topics previously mentioned >?**

**If yes, have these been from your care coordinator; support/key worker; friends; family; GP/ other professionals?** Has your CC been helpful? Encouraging? How have they been helpful?

**Effect of illness / diagnosis on social inclusion**

**You talked earlier about this illness, how has it <their term/s> affected you?**

**Do you think that having a diagnosis of < their term > has had an effect on your social inclusion/your ability to** **< insert previously mentioned topic >**

If yes, go into more detail about influence of diagnosis

**How did you feel about receiving a diagnosis of < insert diagnosis >?**

If client doesn’t feel mental illness has affected their social inclusion/ affected any topics they have brought up, explore other factors they feel has done (e.g. financial/ other commitments)

**Has it become easier over time?**

**Do you feel your life would have been different had you not become mentally unwell? If so, how?**

**Perceptions of others**

**Do you think people generally understand what < insert diagnosis > is?**

**If yes, or no, what makes you say that? Can you give any examples (observed or experienced)?**

Role of family, if not or difficult, delve further, why do you think this is? Are family aware you’d like to do more?

**Feeling more included**

**What do you think could be done to help you feel more socially included, or be able to participate in those areas as you mentioned earlier?** What could friends/family/partners do?

*Hint - can refer to these directly*

**Would you like a greater input to help with** **< topic > from your; care coordinator; support/key worker; friends; family; GP; other professionals?**

**Do you think that your care team/ the professionals who work with you are aware of the importance of social inclusion?**

**Have they discussed social inclusion with you? Do you talk to them about these issues**? Give examples if necessary, e.g. money, relationships, work, accommodation

**If yes, do you discuss these things with them often? Do you feel that they provide you with information and support available in the community?**

**Thank you very much for taking the time to speak with me today.**

**Topic guide for staff focus groups**

**(v.1 17.09.15)**

Background

Welcome and thank you very much for your participation today. My name is < insert researcher name > and I’m a researcher at St George’s University of London.

As you may know, we are conducting research to explore social inclusion in individuals with severe mental health problems, using a measure of Social Inclusion (SInQUE) in order to identify which individuals and groups with mental health problems may be most at risk of social exclusion, in order to develop more targeted interventions to improve health and social outcomes.

We are conducting focus groups with health professionals to explore the nature and of and reasons for lack of social inclusion, develop training and to begin to model an intervention to improve social inclusion. A dedicated website providing access to the measure, training guidelines and guidance in the use of the SInQUE will be created at the end of the study.

I hope you have had the opportunity to review the SInQUE, we have provided copies for your reference.

I would like to reassure you that all of your responses are confidential. Your participation in this focus group is completely voluntary and if at any time during the group you wish to stop, please feel free to let me know.

Do you have any questions before we begin?

Acceptability

1. Do you think the SInQUE is fit for purpose?
2. Do you think the SInQUE is a suitable length and that you would have time to make use of it in your daily practice?
3. Are there any questions/ topics you feel are inappropriate/ irrelevant?
4. Are there any aspects of adult social care that you feel are not yet adequately reflected in the SInQUE?

*Prompt: Would you add/ take away any questions or topics to the SInQUE?*

Application

1. How do you think the SINqUE can best be used to support and improve the social care of adults with mental health problems?
2. Which health professionals do you think would most benefit from this tool?
3. Is there a service user group you feel would most benefit from this tool?

Barriers

1. Can you identify any barriers to the effective application of the SInQUE in your daily practice?

*Prompt: What are the possible solutions to this?*

1. Can you give examples of what you are able to do in your current practice to improve service user’s social inclusion?

Training

1. What do you believe are the training requirements for this tool?

*Prompt: Type of training, resources required and suitability of an online training program?*

Is there anything else that you would like to add?

Thank you very much for taking the time to talk with us today. We really appreciate you sharing your views and taking the time to participate in our study.
