# Supplementary material for: Barriers and facilitators to social inclusion among people with severe mental illness: A qualitative study
Source: PLOS Ment Health. 2025 Apr 24;2(4):e0000137. doi: 10.1371/journal.pmen.0000137 (PMC12798190; doi:10.1371/journal.pmen.0000137)
Supplement: Attachment — Submitted filename: Response to reviewers.docx [file pmen.0000137.s003.docx]

**Response to reviewers: *‘Barriers and facilitators to social inclusion among people with severe mental illness: a qualitative study.*’**

**Journal Requirements:**

1. Please amend your detailed Financial Disclosure statement. This is published with the article. It must therefore be completed in full sentences and contain the exact wording you wish to be published.
2. Please clarify all sources of funding (financial or material support) for your study. List the grants (with grant number) or organizations (with url) that supported your study, including funding received from your institution.
3. State the initials, alongside each funding source, of each author to receive each grant.
4. State what role the funders took in the study. If the funders had no role in your study, please state: “The funders had no role in study design, data collection and analysis, decision to publish, or preparation of the manuscript.”
5. If any authors received a salary from any of your funders, please state which authors and which funders.

*Response:* We have now amended the financial disclosure statement to include the above details: *‘The paper reports work funded by the National Institute for Health and Care Research, School for Social Care Research (NIHR SSCR). The grant reference is C088/T14-035/SGGM-P68 (GM, HK, BLE, JB, and IH). The funders had no role in study design, data collection and analysis, decision to publish, or preparation of the manuscript.’*

6. We note that you have indicated that there are restrictions to data sharing for this study. For studies involving human research participant data or other sensitive data, we encourage authors to share de-identified or anonymized data. However, when data cannot be publicly shared for ethical reasons, we allow authors to make their data sets available upon request. For information on unacceptable data access restrictions, please see <http://journals.plos.org/plosone/s/data-availability#loc-unacceptable-data-access-restrictions>.

 a) If there are ethical or legal restrictions on sharing a de-identified data set, please explain them in detail (e.g., data contain potentially identifying or sensitive patient information, data are owned by a third-party organization, etc.) and who has imposed them (e.g., a Research Ethics Committee or Institutional Review Board, etc.). Please also provide contact information for a data access committee, ethics committee, or other institutional body to which data requests may be sent.

b) If there are no restrictions, please upload the minimal anonymized data set necessary to replicate your study findings to a stable, public repository and provide us with the relevant URLs, DOIs, or accession numbers. Please see <http://www.bmj.com/content/340/bmj.c181.long> for guidelines on how to de-identify and prepare clinical data for publication. For a list of recommended repositories, please see <https://journals.plos.org/plosone/s/recommended-repositories>. You also have the option of uploading the data as Supporting Information files, but we would recommend depositing data directly to a data repository if possible.
 Please update your Data Availability statement in the submission form accordingly.

*Response:* We have now modified the data availability statement to state: *‘Due to the sensitive and potentially identifiable nature of these interviews, select data used and analysed during this study are available from the corresponding author on reasonable request.’*

**Additional Editor Comments (if provided):**
Interesting article and great to see perception of people with lived experience included. In addition to the reviewer's comment, the authors are requested to address the following points:

1. Methods: Diverse and inclusive effort has been taken for coding framework development but more clarity is required in terms of actual coding of the interviews. Did the other authors (line 153-155) code only one common interview/FGD or was there different interview/FGD each? If it was the former, did the authors conduct Inter-rater reliability (IRR). Please report that than just mentioning "high level of agreement was observed"

*Response:* We have now clarified that each member of the team coded one different interview/focus group transcript each: *‘This preliminary framework was shared with five members of the research team along with one different interview/focus group transcript each.’* We did not conduct inter rater reliability during the coding process, in line with Braun and Clarke’s (2021, 2022) guidance on conducting reflexive thematic analysis. They highlight that the reflexive nature of thematic analysis emphasises that researcher subjectivity as an analytic resource rather than a potential source of bias, and that there is no one ‘right’ way to code the data. They discourage focusing on inter-rater reliability. Considering this, we opted to include multiple perspectives in the analytic process (as described in the analysis section) with each member of the team initially analysing a different transcript to encourage a range of insights as we developed the coding frame. Whilst we observed few differences of opinion about the individual codes that were developed, we did not assess inter-rater reliability of the allocation of text to codes by the researchers for the reasons above. We have clarified this with the statement: *‘At this stage, a high level of agreement on the suitability of the coding framework was observed across all members of the team.’*

2. Methods: The first author coded all the interviews and FGDs. Were the interviews used for code development coded again? Can the authors add if there were any efforts to get multiple perspective during the analysis phase.

*Response:* Yes, the interviews used for code development were re-coded by SE once the coding framework was analysed. This is stated in the final statement of the data analysis section, which reads: ‘*SE then used this coding framework to analyse all 15 interviews and 4 staff focus groups’.*

Multiple perspectives from six members of the research team (including the lead author) were integrated at all stages of the development of the coding framework, before this was used in the final analysis. The description of how multiple perspectives is described in the data analysis section, which specifies: *‘Each individual independently coded their respective interview/focus group transcript using the preliminary framework and noted their suggested modifications to the coding framework initially developed by SE. The members of the team involved in coding met to discuss these proposed changes, and SE adjusted the coding framework integrating their feedback. This modified framework was distributed to the same team members, with a different transcript to analyse. Again, the team independently analysed one interview/focus group transcript each using the modified framework and suggested further minor alterations. At this stage, a high level of agreement was observed across all members of the team. SE integrated these suggestions into a finalised coding framework, which the team reviewed and approved.’*

3. Ethics: Based on what the authors report on the result section, there were service users with suicidal ideation. Please clarify how the team handled these situations. Was there a referral service in place for such identified cases?

*Response:* During the consent procedure for the study, we asked service users for permission to inform their GP or care coordinator and request additional support if this was deemed necessary due to participant distress or any immediate safety concerns. Further, due to the nature of the questions asked, acute distress was not anticipated from service users in response to the questions, and no service users became distressed during the course of the interviews. We have added two sentences to the ‘data collection’ section to clarify this: *‘During the consent procedure, we asked service users for permission to inform, and if necessary, obtain support from their GP or care coordinator if a participant were to become distressed or if there were immediate safety concerns. During the interviews, although a few participants mentioned previous suicidal ideation in the context of social exclusion, there were no present serious safety concerns requiring researchers to follow the safety protocol.’*

4. Results: If there are more details on the demographic details of the service users (Table 1), consider adding that. E.g. how many years since diagnosis and in treatment. Were they all taking medication, counseling or both? This additional details would help to situate the findings.

*Response:* We do not have these details available for service user participants. We did not plan to request these details and therefore did not get ethical approval to collect this data. All the participant characteristics data we do hold are reported in the paper.

5. Results: Table 2. Additional demographic details would be helpful. e.g. gender, years of experience, ethnicity

*Response:* Unfortunately, as above, we do not have these details available for staff participants.

6. In line with what other reviewers mentioned, please restructure the result section for better readability. Table 3 can be revised based on how you present your discussion across three levels. Is there a framework across these themes can be developed through your findings describing pathways of mental illness and social inclusion.

*Response:* Thank you for this suggestion. We believe it necessary to present the quotes embedded in the results alongside the relevant section of text, to illustrate the origins of the findings within the interviews and to bring the text to life with the participants’ voices. We have now amended the results section to improve readability, by putting an extra line space between the text and the subsequent quote. We are happy to consider additional recommendations as to how readability can be improved, if this is not sufficiently clear. We agree that the levels of impact are a useful lens through which to view the study themes; we have edited table 3 accordingly. We believe that the micro, meso, macro distinctions provide a deductive framework in which to locate our inductively derived themes. This is in line with the content of the seminal book on social inclusion in mental health (Boardman, Killaspy, & Mezey, 2023).

**Reviewers' comments:
Reviewer 1**

This is an important article outlining the barriers and facilitators for social inclusion for people living with mental illness. The study presents original research with qualitative findings from another study. The qualitative analysis and conclusions are consistent with data reporting for qualitative research and therefore meets the criteria of research integrity and credibility. Of particular relevance is attention to both micro, meso and macro level factors contributing to service user experiences.

1. One area that is not as clear is the section on limitations and strengths which suggest that bias of reporting may have influenced the findings. However, in critical qualitative research such as reflexive thematic analysis this is not necessary the case because you would have "controlled" for this given the multiple perspectives and heterogeneity of the samples. Also the mental health providers are experts because they know these experiences intimately as do the service user participants. The recommendation then maybe to re consider the word "bias" and replace this with ways that the participants were relexive about their participation?

*Response*: Thank you for highlighting this. We have now removed the word ‘bias’ from the strengths and limitations, and have reworded the section to be more in line with the qualitative methodology of the paper: *‘The response rate to this original study was excellent, promoting the likelihood that a range of views from mental health service users within the two study boroughs are reflected. The inclusion of service users with different mental health diagnoses and of staff working in a variety of mental health roles is also a strength of the study, as the study findings are based on a variety of experiences.’*

2. Please also consider providing a reference for thematic analysis in Data analysis section (line 148). I was unable to see a reference for thematic analysis? perhaps I missed this. If using Bruan and Clarke 2022- Thematic Analysis a practical guide suggests that anlysis is interpretive where roles of the researcher is value laden not external to meaning making.

*Response:* Thank you for highlighting this omission. We have now amended this by adding the appropriate thematic analysis reference (Braun & Clarke, 2022) to the methods section.

3. The second area of great importance is the discussion about ethnic minorities more likely to be diagnosed with mental illness - there is one reference for this and is this from the UK? There was a study done in Canada that did not find this to be the case: Rotenberg M, Tuck A, McKenzie K. The role of ethnicity in involuntary psychiatric admission in Toronto, Canada in clients presenting with psychosis. Psychosis. 2019 Jul 3;11(3):273–6. DOI: 10.1080/17522439.2019.1612461 /other studies also suggest that ethnic minority groups that are racialized are less likely to access mental health services due to stigma, social isolation and belonging generally, and specifically during COVID-19 pandemic. This point may require futher articulation and or reflection to unpack the nuance and contradictions.

*Response:* The two papers we cite suggesting that (i) people from ethnic minorities are more likely to be diagnosed with psychosis, and (ii) experience more inequalities in pathways to care, are both recent, high quality systematic reviews with meta-analyses. One of these reviews included findings from 28 studies, while the other included findings from 40. Further, both of these reviews were specifically conducted in English populations, which we felt was the appropriate reference population given that our study was conducted in England.

Overall this article reads well and adds an important body of knowledge to research implications and practice, particularly on barriers and facilitators for inclusion and belonging for people living with mental illness and who carry stigmitizing diagnosis.
Thank you for the opportunity to read this important work!

*Response:* Thank you for your helpful review.

**Reviewer 2**

Thank you for the opportunity to review this interesting and well-written paper. I appreciate that the authors have included perspectives from both individuals with lived experience and mental health professionals.
Methods

1. On page 5, line 87, I recommend including the definition of severe mental illness much earlier in the paper (in the introduction). Additionally, please provide a reference for this definition to clarify how the term was operationalized and how you arrived at this specific definition.

*Response:* We have now included a definition of severe mental illness to the second paragraph of the introduction, with references: *‘Severe mental illness refers to a psychiatric diagnosis that results in considerable role impairment in one or more life domains (e.g., social relationships, employment, independent living) and an extended period of time receiving support from mental health services’.*

2. On page 5, line 90, it would strengthen the paper to include an argument for why interviews were conducted with service users while focus groups were held with staff. Please explain the rationale behind choosing these specific methods for each group, with references.

*Response:* In our experience from previous studies, we have found that individual interviews with service users with severe mental health problems tend to work better than focus groups in terms of enabling people to speak more openly about personal experiences. Additionally, the logistics of trying to bring a group of service users who do not know each other together for a focus group are often too complex to make it a viable option, especially when this study was conducted (i.e. when everything was done face-to-face). However, for busy mental health staff, who often already have an established working relationship, focus groups provide an efficient mechanism to encourage collaborative discussion and gain varied perspectives in a relatively short space of time. We have added brief clarification of this to the ‘study design’ section: *‘We carried out in-depth, individual interviews with service users to enable participants to speak openly about their experiences. We conducted focus groups with staff to encourage varied perspectives and collaborative discussion. We used semi-structured topic guides for the interviews and focus groups.’*

3. Furthermore, it would be helpful to address why staff participants were not remunerated for their participation. This could add important context to the study's design and ethical considerations.

*Response:* Staff participants were not renumerated as they were recruited directly through services and they took part in the focus groups during their regular work time. Thus they were being paid by their employer, as research participation is considered to be a component of staff’s role in the NHS Trusts that take part in such research.

Results

4. Please provide an explanation for the varying numbers of participants in the focus groups. For instance, can a group of only three individuals be considered a focus group? Clarifying this point will aid in understanding the data collection process.

*Response:* We aimed to recruit a sufficient number of staff to enable a group discussion without there being too many participants to not get to hear everyone’s perspectives. We remained flexible on the exact number we aimed to recruit within each focus group, owing to the challenge of scheduling with multiple, busy staff members during work hours. The varied numbers within focus groups therefore reflects this scheduling challenge – some potential participants were unavailable for the dates and times chosen and others had to drop out last minute. This meant that some focus groups had fewer participants than others. In spite of this, we found that the discussions in all four focus groups were rich, collaborative, and valuable, regardless of the number of participants in each.

5. I suggest mentioning somewhere in the results or methods sections any steps taken to address situations involving suicidal participants. This is crucial given the nature of the study population, and how someone mentioned this specifically in the results. Where there any steps taken to give this person support? This is important in highlighting ethics in undertaking research with “at-risk” populations.

*Response:* During the consent procedure for the study, we asked service users for permission to inform their GP or care coordinator and request additional support if this was deemed necessary due to participant distress or immediate safety concerns. Further, due to the nature of the questions asked, acute distress was not anticipated from service users in response to the questions, and no service users became distressed during the course of the interviews. We have added two sentences to the ‘data collection’ section of the methods to clarify this: *‘During the consent procedure, we asked service users for permission to inform, and if necessary, obtain support from their GP or care coordinator if a participant were to become distressed or if there were immediate safety concerns. During the interviews, although a few participants mentioned previous suicidal ideation in the context of social exclusion, there were no present serious safety concerns requiring researchers to follow the safety protocol.’*

6. The example regarding cultural backgrounds is interesting. It would be valuable to expand on this section and in the discussion as well and provide additional examples to give a more comprehensive view of the cultural factors at play.

*Response:* Thank you for this reflection – we agree that this is a very interesting point. We did not note additional instances within the data where participants directly reflected on the role of their cultural background, so we are unable to provide additional examples of this.

7. The concept of "sufferer" is being mentioned several times by the participants. It will be interesting to unpack this where you talk about (internal) stigma in the results and discussion. Consider elaborating on this term and how it is perceived or used within the context of the study, and how it re-enforces self-stigma.

*Response:* We have added a line to reflect on the use of this term and how it may reflect self-stigma to the discussion: *‘Many participants referred to themselves as ‘sufferers’, which may be an additional indicator of self-stigma.’*

8. On page 24, line 434, the term "A&E" is used. For clarity, please define this abbreviation for an international audience who may not be familiar with the term.

*Response:* Thank you for flagging this – we have now included the definition for this abbreviation on first appearance.

Discussion

9. In the discussion section, you touch upon perceptions at the organisational level. However, these aspects are not reflected in the results. Were there any findings related to organizational perceptions that could be integrated into the results section? If so, including them would provide a more comprehensive discussion of the topic.

*Response:* We have reflected on any organisational level factors that were present in the findings within the theme ‘system of mental healthcare and support’. These primarily related to staff relationships with service users and their knowledge of opportunities, the resources available for support, and the broader priorities available within the system. We did not find any additional reflections relating to organisational perceptions.

10. A discussion of these organisational level barriers and facilitators more in-depth in the discussion can be added as well to strengthen the paper and give more credibility to the ecological perspective take that the authors suggest in the beginning of the results.

*Response:* We have now expanded on our discussion of the levels of influence of barriers and facilitators to social inclusion, including more detail about the factors that operate at each level: ‘*Our findings show that social inclusion among people with severe mental illness is a complex concept that can be influenced by factors at all levels of society. Results concur with the theoretical drivers of social exclusion as described by Boardman et al., in that barriers and facilitators to social inclusion can operate at macro, meso and micro levels. At the micro level, social inclusion can be influenced by an individual’s mental health, beliefs, internalised stigma, self-confidence, and coping styles. At the meso level, influences include an individual’s social network, and their mental health care team, and community resources. At the macro level, factors such as the wider health and social care system, socioeconomic and political factors, and societal attitudes can act as social inclusion barriers and facilitators.’*

11. The ecological perspective take can also be integrated more in the introduction. For example, from what is known in the literature, have factors at these different levels been researched? Which levels have been researched more? Is there a gap in a specific level? Do the factors interact across levels?

*Response:* We have highlighted the examples where we are aware that these barriers at different levels have been researched previously and added more detail about the levels of impact such factors may operate across: ‘*Several possible barriers to social inclusion in this population that operate across multiple levels of influence have been proposed, including the nature of mental health symptoms themselves, poor self-esteem, low confidence, discrimination, and lack of opportunity. These factors operate across micro (individual), meso (groups and relationships), and macro (societal) levels of influence’.* We felt that further expansion on these levels of influence within existing literature was most usefully addressed in the discussion alongside our own findings, as we have outlined in point 10.

**Reviewer 3**

The manuscript investigates the barriers and facilitators to social inclusion among individuals with severe mental illness (SMI) through qualitative research. The study employs individual interviews with service users and focus groups with mental health staff, drawing participants from two NHS trusts in London. Six primary themes emerged from the inductive thematic analysis: individual characteristics, the role of mental illness, social relationships, the mental health care system, financial situation and opportunities, and stigma. I have only a few comments and suggestions:

1. The study includes interviews with only fifteen service users and four focus groups with sixteen staff. While this may be sufficient for qualitative exploratory research, a discussion about the sample size’s adequacy concerning the breadth of the research question would strengthen the paper.

*Response:* We believe that the sample of 31 participants is reasonable for a qualitative study of this nature and is consistent with general guidance about sample sizes in qualitative research (Hennink & Kaiser, 2022; Guest, Bunst, & Johnson, 2006). Braun and Clarke (2021) note that one of the key considerations in relation to participant numbers in qualitative research is to consider the depth and richness of the data collected, as a measure of whether additional data collection is justified. As we found our data to be both rich and suitable to answer the research question, we did not find it necessary to recruit additional participants or consider the sample size as a limitation. Furthermore, as a reflection of the breadth of the research question, we sought insights on this question from both service users and staff, using both interviews and focus groups. We believe these varied insights using a variety of data collection methods is a notable strength of the study.

2. More detailed demographic information about the service user participants would be beneficial to understand the context of the findings fully.

*Response:* Please see response to editor comments numbers 4 and 5.

3. The process of thematic analysis should be described in more detail. For instance, how were the themes identified, and what steps were taken to ensure the reliability and validity of the coding process?

*Response:* Thank you for raising this. The themes were identified through a collaborative coding process among the research team, as described in the data analysis section. Note that the coding framework includes within it the higher-level themes. This has been clarified throughout the data analysis section: *‘SE initially coded four interviews and one focus group and developed a coding framework with preliminary themes based on this. […] Each individual independently coded their respective interview/focus group transcript using the preliminary framework and noted their suggested modifications to the coding and thematic framework initially developed by SE. The members of the team involved in coding met to discuss these proposed changes, and SE adjusted the coding framework and themes, integrating their feedback.’*

In relation to the point about reliability and validity, please see response to editor comment number 1.

4. The conclusion drawn about the need for social interventions, individualized care, and stigma reduction is pertinent. However, it would be useful to provide specific recommendations or examples of potential interventions or strategies that could address these issues. This would help bridge the gap between research and practice.

*Response:* We have mentioned potential interventions that we are aware of for social outcomes (Community Navigator programme, SCENE programme, Connecting People intervention), individualised care (the SInQUE), and stigma reduction (targeted and population level anti-stigma campaigns) in the ‘implications for research’ and ‘implications for practice’ sections of the discussion. We have added a summary of the evidence relating to this, with reference to two reviews on further possible social interventions for people with mental illness: *‘While a wide range of interventions relevant for social inclusion related outcomes have been developed, the current evidence base is not large and the most effective types of care and support remain to be established in many areas [45,46]. This represents an important research priority.’*

**Reviewer 4**

Overall: This is a robust study that highlights barriers and facilitators of social inclusion in people with mental ill health. The authors have conducted a qualitative study that highlights the multi-faceted issues that impact on people’s experiences from the perspective of key stakeholders consisting of service users and staff. With some amendments and refining, this could add to the literature on social inclusion in mental health. the best ways to help with this need are not well understood
General comments:

1. There appears to be some conflation between social inclusion and exclusion in the sense that it’s not clear if they are used interchangeably or for specific points? It would be good to clarify this use with a definition and/or stick to use of the one.

*Response:* Thank you for highlighting this. We have now provided clear definitions of both social inclusion and exclusion at the start of the introduction: *‘Social exclusion refers to an individual’s lack of opportunity to experience the typical rights, relationships, and activities available to most people in society, typically for reasons that are beyond their control. Social inclusion, meanwhile, is defined as having access to social and economic opportunities, and a sense of community belonging. It is a multi-dimensional and dynamic concept, that has been conceptualised to encompass five key domains: productivity (including education and employment), social integration (including social relationships and networks), consumption (including poverty and financial wellbeing), access to services (including primary care), and political engagement (including voting and campaigning).’*

2. The format of the results section is a bit hard to read could the quotes be structured in a way they are separate from main text

*Response:* We have now amended the results section to improve readability, by putting an extra line space between the text and the subsequent quote. We are happy to consider additional recommendations as to how readability can be improved, if this is not sufficiently clear.

3. There are a lot of subthemes in the results and these need to all be contextualised in the discussion section. At current, the discussion section is not detailed enough. There is more detail in implications but this should be higher up in the discussion

*Response:* We did not feel that it is feasible to summarise and contextualise every subtheme individually in the discussion, as this section would become very long. Instead, we have tried to contextualise findings at the level of themes, and have added additional detail for subthemes that we felt particularly needed elaboration.

4. At the moment, it’s not clear how social inclusion and exclusion are different for different mental health problems. Literature highlights the differences in agoraphobia for example in people with psychosis and this is an important thing to include in both the discussion and implications. It should be reported on how the themes are more/less relevant for different problems.

*Response:* Our aim with this study was to reflect on the experience of social inclusion for individuals with a severe mental illness, rather than comparing experiences according to specific diagnoses. This is reflected in our findings, where participants did not tend to reflect on the specifics of their diagnoses, but rather discussed their experience of mental ill health more broadly. We did not find specific differences in people’s experiences of social exclusion according to diagnoses. We reflect on this in the limitations: ‘*The inclusion of service users with different mental health diagnoses and of staff working in a variety of mental health roles is also a strength of the study, as the study findings are based on a variety of experiences. However, this does limit our understanding of the extent to which these findings may relate to any specific diagnostic group or service setting.’*

5. Line 693: The sample had an appropriate representation of ethnic minorities, suffice to the population of the UK which is a strength. It would be good to do a sub-analysis of factors that might differ in ethnic minority groups/.

*Response:* We have carefully reported in the results all instances where participants did reflect on their ethnicity and/or their culture. We have acknowledged this in the limitations, as we note it is possible that additional insights were missed: *‘Although service user participants were well balanced in terms of gender, the majority were of White ethnicity. This may mean that important insights that are particularly relevant for mental health service users from ethnic minorities were missed.’*

6. Line 23: “the best ways to help with this need are not well understood “ This line of the abstract could be worded differently to make it clearer.

*Response:* We have rephrased this sentence to hopefully be clearer. It now reads: *‘Further, it has not been clearly established how to improve social inclusion in this population.’*

7. Line 42: Using the term occur suggests it’s a random event – could you expand on social inclusion a bit more? Including a definition of social inclusion/exclusion would be good here.

*Response:* Thank you for this suggestion, we agree that these definitions can be improved. We have, therefore, removed ‘occur’ from the description, and instead have expanded more clearly on our definitions of these terms: *‘Social exclusion refers to an individual’s lack of opportunity to experience the typical rights, relationships, and activities available to most people in society, typically for reasons that are beyond their control. Social inclusion, meanwhile, is defined as having access to social and economic opportunities, and a sense of community belonging.’*

8. Line 50-54: This is a very long sentence, could it be split into two or summarised?

Response: We have hopefully made this sentence easier to follow, by making it more concise and dividing it using a colon: ‘*From the perspective of key mental health stakeholders, including people with lived experience of serious mental illness, the key social inclusion goals for this population include: participation in social activities, housing, community involvement, employment, health, and service utilisation*.’

9. Line 60: Does productivity and social integration reduce or could a better term be used?

*Response:* The authors of the study we cite these findings from used the term ‘reduce’ to describe their findings, hence why we have used this term.

10. Line 73: why is it needed? What is the gap from previous research?

*Response:* We have now clarified this: ‘*However, there is a lack of in-depth research examining the barriers and facilitators to social inclusion according to people with lived experience and mental health professionals.*’

11. Line 79: Long sentence – check grammar too.

*Response:* We agree that this sentence was hard to follow. We hope we have now clarified this: *‘Such insight can help inform the development of relevant and targeted support for social exclusion and promote understanding of key aspects of recovery for service users themselves. These principles are in line with recovery-oriented practice.’*

12. Line 86: Check person and sentence starter.

*Response:* We have rephrased this sentence to read: *‘We conducted a qualitative study to explore service user and staff views on the barriers and facilitators to social inclusion among people with severe mental illness.’*

13. Line 94: Sample –

*Response:* We have now changed the title to: *‘Sample and recruitment’.*

14. Line 116: were service users involved in the development of the topic guide?

Response: Service users were not involved in the initial design of the topic guides, however we consulted two service user research groups to advise on the draft of the topic guides and modified these according to their feedback. We have now clarified this with additional detail: *‘The topic guides were reviewed by two service user research advisory groups and a stakeholder advisory group from the larger SInQUE study and were refined according to their feedback.’*

15. Line 139: Is there a reason why the interviews were conducted so long ago and results reported now? Has research since then been reviewed in light of this?

*Response:* The interviews were unable to be analysed in the timeline of the original project, due to time restraints during that project. The analysis was revisited during a later project, during which there was time available for this. The paper was written at the same time as the analysis, so the literature included throughout is up to date and reflects the latest research. Please note we have reflected on the potential limitations of the gap between when the interviews were conducted and the analysis in our limitations: ‘*It is important to note that these interviews were conducted between 2016 to 2017, before the COVID-19 pandemic. Therefore, important nuances relating to social exclusion that were related to this period are not represented in this study.*’

16. Line 149: Were the interviewers involved in the transcript analysis? It looks like they were different members of the team so would be good to consider this limitation in the analysis process?

*Response:* The interviewers were not involved in the data analysis process. The interviewers had moved on to new posts at the time of analysis and thus were not able to participate directly in data analysis, however both are co-authors of the paper and have reviewed and approved the findings. We have added a sentence to the limitations in acknowledgement of this: *‘Finally, the original interviewers were not involved in the analysis process. Therefore, results have not benefitted from any additional insights gained through direct experience of interviewing participants.’*

17. Table 1: It’s great to see the majority of the sample had diagnosis of a psychotic disorder which is a difficult population to recruit – were any sub-analyses conducted for this group to see if specific barriers and facilitators applied based on diagnosis?

*Response:* We did not reflect on findings according to specific diagnoses, in line with the study aim. Please additionally see response to comment 4.

18. Table 3 would benefit from greater detail in the caption

*Response:* We have now edited the caption to provide more detail: *‘Themes and subthemes reflecting barriers and facilitators to social inclusion.’*

19. Line 201: it would be good to support language difficulties with service user quotes if this applied to them too? did it affect those for whom English wasn’t their first language? Important to note this because majority of the sample was White.

*Response:* We agree that this is an important point, however service user participants in our sample did not reflect on language difficulties themselves; this was a point that was only raised by staff participants. Therefore, we do not have participant quotes to illustrate this point from their perspective.

20. Line 216: ‘certain’ – who does this pertain to?

*Response:* We have changed this to ‘some’.

21. Line 307: It’s important to state if this is coming from a specific demographic – assuming this isn’t the case for everyone but the specificity is important to state if it is explicitly coming from one group. Line 304 says ‘a few’: was this explicitly non-white participants or was this also white participants?

*Response:* Thank you for highlighting this. We have now revised this sentence to be more precise, as on reflection this specific point only related to one service user’s views. The sentence now reads: ‘*One service user participant of Black ethnicity noted an additional challenge imposed by the cultural background of their family and how this influenced their perception of and response to their mental illness*.’

22. Line 419: does this issue come under finances rather than systems of support? The other points made in this subtheme are suggestive of support from them whereas the subject of budgeting and time maybe comes under finance systems?

*Response:* This point about budgeting, caseloads, and staffing relates to the pressure within the mental healthcare system on staff and how staff viewed this as a barrier to supporting service users with social inclusion. The theme titled ‘finances and access to opportunities’, meanwhile, relates to the burden of financial stress and limited opportunities experienced by service users, and how this can prevent social inclusion. We therefore believe that issues relating to staffing budgets, caseloads, and shortages is most appropriately located within the theme of ‘system of mental healthcare and support’ rather than the theme of ‘finances and access to opportunities’.

23. Line 490: was stigma more of an issue for psychosis patients? I can see this is expanded on further down in relation to healthcare but it would be good to expand on the personal relationships too

*Response:* The theme of ‘stigma’ was observed throughout all interviews. We did not assess patterning of themes according to diagnosis, in line with the aim of the study (please see response to comment 4).

24. Line 567: Appreciate the micro, meso, macro but I wonder if a framework might be better to highlight the hierarchy of influence.

*Response:* We believe that the micro, meso, macro distinctions provide a deductive framework in which to locate our inductively derived themes. This is in line with the content of the seminal book on social inclusion in mental health (Boardman, Killaspy, & Mezey, 2023).

25. Line 576: I suggest the suggestions for solutions should move to the end

*Response:* Thank you for this suggestion – we have now moved this paragraph to the end, as suggested.

26. 582: if they are both barriers and facilitators, could you expand on what turns them from facilitators to barriers? E.g. addition of or lack of xyz in individual family relationships.

*Response:* We have now clarified this with the factors that may make relationships barriers or facilitators: *‘Findings suggest that social, familial, and romantic relationships for this group can act as both barriers and facilitators to their social inclusion, depending on whether support, understanding, and encouragement were absent or present in these relationships.’*

27. Line 665: reference needed

*Response:* Thank you for this suggestion; we have now added a reference for this line.
